# Human cranial stem cells: developmental, pathologic, and therapeutic implications

**DOI:** 10.3389/fcell.2026.1718526

**Published:** 2026-02-12

**Authors:** Anvith Reddy, Anna Means, Sarah Qaddo, Victoria Tong, Franklin Gergoudis, Noah Alter, Ricardo Torres-Guzman, Michael Golinko, Wesley Thayer, Izabela Galdyn, Galen Perdikis, Matthew E. Pontell

**Affiliations:** 1 Vanderbilt University, Nashville, TN, United States; 2 Department of Plastic Surgery, Vanderbilt University Medical Center, Nashville, TN, United States; 3 Department of Plastic Surgery, Monroe Carell Jr. Children’s Hospital at Vanderbilt, Nashville, TN, United States

**Keywords:** cranial development, craniofacial regeneration, craniosynostosis, regenerative therapeutics, skeletal stem cells, stem cell signaling, tissue engineering

## Abstract

Cranial skeletal stem cells are central to skull development, maintenance, and repair. These stem cell populations balance self-renewal with lineage commitment, providing osteogenic, chondrogenic, and stromal outputs required for craniofacial growth. While bone grafting remains the gold standard for reconstruction, limitations in donor supply and morbidity have driven interest in harnessing endogenous regenerative programs. In this review, we synthesize current knowledge of human cranial stem cell biology, drawing on developmental, molecular, and imaging data. We delineate their distinct niches within the sutures, dura, and periosteum, as well as the signaling pathways that regulate their function. We then highlight future avenues of investigation, including high-resolution profiling of human stem cell populations and development of mechanism-based regenerative strategies that integrate cell therapy with scaffold design.

## 1 Introduction

Each year, reconstructive surgeons perform over 50,000 craniofacial procedures to address congenital abnormalities and head and neck defects. This high demand is reflected in the global bone graft and substitutes market, which may exceed $4 billion by 2030 (Bone Grafts And Substitutes Market Size, 2025; Plastic Surgery Statistics, 2025). Autologous bone is the gold standard for craniofacial reconstruction. However, obtaining it can be difficult due to substantial donor-site morbidity or limited donor-site availability. Tissue engineering offers a promising path to overcome these limits by leveraging regenerative processes that combine cells, scaffolds, growth factors, and gene therapy. Skeletal stem cells, with their ability to regenerate bone and form all required lineages, represent an ideal candidate for these strategies (Matsushita et al., 2020).

Despite many advances in murine skeletal stem cell biology, data on human cranial SSCs remain sparse. Most foundational principles of SSC identity and signaling come from long bone or mouse studies. Increasing evidence points to key regulators in humans that have not been identified in mouse research, such as CADM1, FOXP1/FOXP2, ZEB2, and microRNA-146a (Ambrosi et al., 2025; Cheung et al., 2014; He et al., 2021; Hochmann et al., 2023; Tani et al., 2023). This suggests that human cranial SSCs operate under distinct transcriptional and epigenetic programs. Direct experimental evidence remains limited by the scarcity of healthy human tissue, the inability to perform lineage tracing, and reliance on specimens obtained from surgical correction of disease. Key aspects of human cranial SSC biology are still mostly inferred rather than directly observed.

In this review, we synthesize current knowledge of human cranial SSC identity, localization, and molecular regulation, and explicitly outline the unanswered questions and methodological constraints that shape the field. We define what is currently known and highlight what remains unresolved. Further, we delineate the critical areas of investigation needed to advance mechanism-based, human-specific stem cell therapies for craniofacial reconstruction.

### 1.1 Operational definitions and terminology

Throughout this review, we use the term skeletal stem cell (SSC) as a functional designation rather than a universally validated cell identity. In its strictest sense, an SSC is defined by: (1) long-term self-renewal, (2) multilineage differentiation capacity, and (3) *in vivo* contribution to tissue maintenance or regeneration. In murine systems, these criteria are established through lineage tracing, clonal analyses, and genetic perturbation studies. In humans, direct lineage tracing and long-term *in vivo* assays are not feasible, so most studies rely on surrogate evidence, such as surface marker expression, transcriptional state, spatial localization, and *in vitro* assays. We use the term progenitor to describe cranial mesenchymal populations that exhibit limited lineage potential, transient expansion, or differentiation capacity. Lastly, the term mesenchymal stem cell (MSC) is reserved for populations defined operationally by plastic adherence, tri-lineage differentiation *in vitro*, and immunophenotyping. These properties do not necessarily equate to *bona fide* skeletal stem cell function *in vivo*.

## 2 Calvarial development and structure

### 2.1 General structure and cellular origins

The calvarium consists of eight bones that protect the brain and provide structural support for the head and face. Four singular bones (ethmoid, sphenoid, occipital, and frontal) and two paired bones (parietal and temporal) comprise the calvarium. The calvarium is further divided into the cranial vault and the cranial base, the latter of which consists of the frontal, sphenoid, ethmoid, occipital, and paired temporal bones. It acts as the articulation between the calvarium (neurocranium) and the facial skeleton (viscerocranium).

Osteogenesis starts between the 6th and 7th weeks of embryonic development and continues into the early to mid-twenties. All bones in the human body originate from three derivatives: the lateral plate mesoderm, somites (paraxial mesoderm), and neural crest cells. Calvarial bone development differs from that of long bones (Doro et al., 2017). Long bones develop from the lateral plate mesoderm, while the axial skeleton develops from the paraxial mesoderm. Long bones undergo endochondral ossification, where cartilage forms and is later replaced by bone. In contrast, the skull develops as a mesenchymal sheet that ossifies directly, without a cartilaginous stage. These differences are mainly linked to differences in cell origin. Long bones develop from mesenchymal cells. Much of the calvarium, however, develops from ectoderm-derived cranial neural crest cells, which migrate from the embryonic neural plate and later become the cranial osteoblasts and osteocytes (Doro et al., 2017).

Most bones of the viscerocranium and neurocranium develop from the neural crest cells, except the temporal and occipital bones (Dash and Trainor, 2020; Gross and Hanken, 2008; Kuratani, 2018; Le Douarin, 2012; McBratney-Owen et al., 2008; Noden and Trainor, 2005). The squamous portion of the temporal bone primarily contributes to the cranial vault. It develops from the neural crest cells, while the remaining portions primarily contribute to the cranial base and develop from the paraxial mesoderm. Similarly, the squamous portion of the occipital bone mainly contributes to the cranial vault. It develops from the neural crest cells, while the remainder primarily contributes to the skull base and is derived from paraxial mesoderm.

### 2.2 Ossification in normal development

The frontal and paired parietal bones ossify by intramembranous ossification. The ethmoid bone ossifies by endochondral ossification. The occipital, sphenoid, and paired temporal bones undergo ossification by both processes (Dias et al., 2020; Jiang et al., 2002; McBratney-Owen et al., 2008). The squamous part of the occipital bone contributes mainly to the cranial vault and forms by intramembranous ossification. The rest contributes to the cranial base and ossifies endochondrally. The same is true for the temporal bone. The squamous and anterior mastoid portions, which form most of the cranial vault, undergo intramembranous ossification. The rest forms by endochondral ossification (Galea et al., 2021; Grzonkowska et al., 2023; McBratney-Owen et al., 2008). In the sphenoid bone, the body and lesser wings form the skull base and ossify endochondrally. The greater wings and pterygoids, which connect to the viscerocranium, ossify mainly by the intramembranous process (Doro et al., 2017). This highlights the complexity of osteogenesis in the calvarium.

The bones of the cranial vault are joined together by sutures (fibrous joints), whereas the bones of the cranial base are joined together by synchondroses (cartilaginous joints). In the setting of cranial disease processes, the sutures are often referred to as the major (metopic, sagittal, paired coronal, and paired lambdoid) or minor (parieto-squamous, fronto-sphenoidal, etc.) (Pontell et al., 2025; Sullivan et al., 2024) sutures. The metopic suture closes between 3–9 months, whereas the remaining major and minor sutures often remain open until the late teenage years or even the early twenties. Cranial base synchondroses are growth centers that predominantly drive the anterior-posterior development of the cranial base and viscerocranium, whereas the sutures are often cited as the major sites of bone growth during craniofacial development (Baer, 1954; Lenton et al., 2005; Menon et al., 2021; Opperman, 2000; Rice, 2008).

As the calvarium develops, bones are surrounded by tissue layers that support cranial growth and the development of vascular, nervous, and lymphatic structures. The periosteum is a thin fibrous membrane covering the outer surfaces of bones, with several roles in cranial bone biology (Maia Ferreira Alencar et al., 2020). These include bone formation and growth, supported by blood supply from the vasculature. The periosteum also aids in bone modeling and repair after injury. It has two layers (Dwek, 2010). The outer fibrous layer provides structural support and protection. It also serves as an attachment point for cranial ligaments and tendons (Li and Fennessy, 2021). The inner cambium layer contains osteoprogenitor cells needed for bone formation and repair. Studies in human and animal models show a high density of vascular and glial elements in this layer, supporting activity during ossification, growth, and regeneration (Debnath et al., 2018; Ferretti, 2014; Roberts et al., 2015). The meninges are deep to the cranial bones and protect the central nervous system. Within the calvarium, the dura mater lies just below the bone, then the arachnoid mater, and finally the pia mater, which closely covers the brain and spinal cord (Abbaoui et al., 2023).

These developmental distinctions in cell origin and ossification mechanisms establish the structural landscape in which cranial SSCs reside. Building on this foundation, we next examine the molecular interplay and localization of stem cell niches.

## 3 Stem cell localization

Before examining the localization and molecular behavior of cranial skeletal stem cells, it is essential to acknowledge key methodological constraints that shape current human data. These limitations are summarized in Box 1. A number of human and animal studies have identified bone as a site of various stem cell populations, including skeletal stem cells (SSCs), in both long bones and the calvarium. The strongest data to date implicates stem cell development in two regions of calvarial bones, one being the sutures between developing calvarial bones and one being in the periosteum (Debnath et al., 2018; Doro et al., 2024; Doro et al., 2017; Li et al., 2021; Li et al., 2023b; Maruyama et al., 2016; Menon et al., 2021; Sun et al., 2025; Zhao et al., 2015).

Box 1 **Methodological limitations in human cranial SSC research**

**Limited access to human tissue:** Due to inherent constrains of obtaining healthy, physiologic human cranial tissue, most human specimens are obtained during surgical correction for craniosynostosis or trauma. This leads to an overrepresentation of pathologic tissue and a relative underrepresentation of physiologically normal sutures.

**Experimental Manipulation:** In contrast to murine models, there is no practical method for *in vivo* lineage tracing or similar manipulation in humans. Therefore, human research must rely on techniques like surface marker staining, *in vitro* assays, or xenografts that may not faithfully report long-term stemness.

**Definitions of SSC populations:** Canonical murine SSC markers like Gli1, Axin2, Prx1 lack direct human orthologous evidence. These markers have not been validated as lineage-defining SSC identifiers in humans as most human studies rely on expression-state inference.

**Niche disruption *ex vivo*:** It is possible to reliably create *in vitro* cultures from human cranial tissue. However, enzymatic digestion and monolayer cell culture cannot fully recapitulate the *in vivo* environment or niche dependencies.

### 3.1 Cranial suture mesenchyme

Cranial sutures are a likely source of SSCs due to proliferation and outgrowth at suture edges during calvarial neonatal and postnatal development (Doro et al., 2017). While a number of papers have investigated the presence of SSCs in calvarial cell populations, the majority of this work has focused on murine models (Doro et al., 2017; Wilk et al., 2017; Zhao et al., 2015). To highlight the additional potential of SSCs in calvarial regions, this topic is briefly outlined below. Combinations of lineage-tracking and gene-mutation analyses have identified cranial stem cells in mice across several models. Vital dye labeling identified a small population of cranial stem cells residing in sutural mesenchyme adjacent to osteogenic fronts (Doro et al., 2017). In another study, markers of SSCs were identified in suture mesenchyme. Mutations in some of these genes (e.g., *Gli1*) led to inhibition of SSC growth and craniosynostosis (Zhao et al., 2015). In a model of suture transplantation and lineage tracing, Wilk et al. (2017) linked suture markers with cells that contribute to calvarial bone defect regeneration.

In humans, direct lineage tracing is not feasible, but other techniques (single-cell profiling, xenograft assays, histological studies, etc.) have identified analogous SSC populations within cranial sutures. These populations include DDR2+ and CTSK+ stem cells, which display conserved properties in both bone regeneration and suture patency (Bok et al., 2023). While human studies have confirmed that the suture mesenchyme harbors multipotent stem cells, there is a strong need to continue refining their mechanistic details (Bok et al., 2023; Li et al., 2021; Li et al., 2023a).

Despite broad agreement that the suture mesenchyme harbors progenitor populations, major uncertainties remain regarding their stability, lineage potential, and true stemness in humans. Murine Gli1+, Axin2+, and Prx1+ populations exhibit self-renewal and long-term contribution to bone, but equivalent lineage-defining markers have not been validated in humans. Consequently, whether human sutural cells function as *bona fide* stem cells or as short-lived progenitors remains a central unresolved question, with substantial implications for how we conceptualize craniosynostosis and therapeutic regeneration.

### 3.2 Periosteal stem cells

Based on the known localization of osteoblasts in the periosteum of cranial bones, Debnath et al. (2018) examined this region for the presence of SSCs using molecular markers. In mice, the periosteum contains cells that fit the definition of stem cells: they are self-renewing and multipotent, able to give rise to multiple bone lineages. Debnath et al. (2018) then identified similar populations of potential SSCs in the periosteum of the human femoral bone. While the putative stem cells were isolated from a long bone (femur), they differentiated *in vitro* into both intramembranous and endochondral structures. Similarly, Ortinau et al. (2019) also used mouse SSC markers to identify potential stem cells in the periosteum of human femoral and tibial bones, again suggesting that the periosteum is indeed a source of SSCs.

While multiple sites have been identified as containing SSCs in calvarial tissue, there appears to be variability in stem cell populations across regions. High-throughput sequencing of mouse and human frontal and parietal compartments revealed a broad spectrum of differentially expressed genes, including genes involved in cell matrix, transcriptional factors, cytokines, and receptors (Homayounfar et al., 2015). Menon et al. (2021) showed that isolated human SSCs from the frontal calvarium had greater osteogenic potential than osteoblasts from parietal populations.

Although periosteal SSCs are well characterized in long bones, far less is known about how periosteal progenitors behave in the unique mechanical and developmental environment of the calvarium. Human periosteal cells isolated from long bones often exhibit robust endochondral potential, but evidence suggests that calvarial periosteum may be more restricted, favoring intramembranous fates. Whether this reflects intrinsic lineage commitment or niche-specific cues remains unclear. This discrepancy underscores the need for cranial site-specific profiling rather than extrapolation from appendicular skeleton data.

### 3.3 Intracalvarial stem cell niches

While the majority of progenitor cell populations in the calvarium are concentrated in the cranial sutures and periosteum, emerging evidence from human and animal studies suggests that these populations may also be present in the endosteal and diploic regions of the calvarial bone itself (Di Pietro et al., 2020). However, the density and functional significance of these intradiploic stem cells appear to be much lower than those in the suture mesenchyme and periosteum, and most studies emphasize the suture and periosteal regions as the major stem cell reservoirs for calvarial bone growth and repair (Debnath et al., 2018; Maruyama et al., 2016).

### 3.4 Molecular regulation of human cranial SSCs

The molecular programs that govern cranial SSC behavior are only partially defined in humans. Available data suggest that SSC maintenance and lineage progression depend on the coordinated activity of multiple developmental pathways, including Wnt/β-catenin, Hedgehog, FGF, BMP/TGF-β, and Eph/Ephrin signaling. Human cranial SSCs also exhibit distinctive epigenetic features. These include site-specific enhancer architectures and chromatin accessibility profiles. It has also been shown that these differ across skeletal regions and correlate with variations in regenerative potential (Li et al., 2023a). These regulatory layers interact to control self-renewal, osteogenic commitment, and sensitivity to inflammatory or mechanical cues within the calvarial niche.

Most mechanistic insight, however, derives from murine perturbation studies. Comparative single-cell and epigenomic analyses demonstrate substantial divergence between mouse and human SSC regulatory hierarchies, with limited conservation of key enhancers and transcriptional networks (Li et al., 2025; Li et al., 2024). As a result, it remains uncertain whether canonical pathways such as Wnt, FGF, or BMP exert equivalent effects on human SSC fate, or whether dura-derived, periosteal, and mechanical cues identified in mice play similar roles in human sutures. These discrepancies underscore that the molecular logic of human cranial SSCs is still largely inferential and highlight the critical need for direct, human-specific functional studies.

Together, these molecular and anatomical insights establish the framework for understanding how perturbations in SSC signaling may underlie disorders of suture biology. The following section examines how syndromic and nonsyndromic craniosynostosis intersect with these pathways to regulate SSC behavior.

### 3.5 Evidence for species and site–specific cranial SSC programs

Single-cell RNA sequencing of human skeletal stem cells from 10 distinct anatomical locations shows significant transcriptional and phenotypic divergence between cranial and long-bone compartments (Ambrosi et al., 2025). Cranial populations exhibit distinct proportions of chondrogenic, osteogenic, stromal, and fibrogenic subtypes when compared to long-bone populations (Ambrosi et al., 2025). Furthermore, these differences can be traced back to embryologic origin. Neural crest–derived skeletal stem/progenitor cells within the sagittal suture are characterized by CADM1 expression and FOXP1/2 transcriptional networks. These populations are fundamentally distinct from mesoderm-derived perichondrial skeletal progenitors of long bones (He et al., 2021). Additionally, cranial stem cell populations exhibit distinct molecular marker profiles, including higher GLI1 and AXIN2 expression (Di Pietro et al., 2020). Taken together, human SSC studies refine murine-derived frameworks of skeletal stem cell biology. There are species- and site-specific differences that might limit direct extrapolation. However, these findings underscore that SSC evidence is synergistic but still incomplete.

## 4 Insights from craniosynostosis into SSC regulation

### 4.1 Clinical overview

Craniosynostosis is a congenital condition characterized by premature fusion of one or more cranial sutures, leading to restricted skull growth, compensatory cranial deformation, elevated intracranial pressure, and, in some cases, neurodevelopmental delay. While the clinical presentation is heterogeneous, craniosynostosis provides a unique window into the molecular regulation of cranial development, as many genes associated with this disorder converge on pathways essential for skeletal stem cell maintenance and differentiation.

### 4.2 Genetic pathways in syndromic craniosynostosis

While most cases of craniosynostosis are nonsyndromic, syndromic forms have been identified as having key genetic drivers of calvarial development, which are increasingly recognized as regulators of SSC biology. Some of these genes have been identified in mice as key regulators of cranial development and craniosynostosis. Despite variation in specific mutations, most syndromic craniosynostosis genes map to a limited number of developmental pathways that orchestrate cell-fate specification, including FGF, BMP/TGFβ, Wnt, Hedgehog, and MAPK signaling.

Most syndromic cases of craniosynostosis are autosomal dominant, suggesting that downregulation of certain genes is critical for normal suture development (reviewed in Bok et al., 2023). Many genes mutated in craniosynostosis regulate the decision between proliferation and differentiation, such as cytokines FGFR1 (Muenke et al., 1994), FGFR2 (Bochukova et al., 2009), FGFR3 (Doherty et al., 2007), EFNA4 (Tung et al., 2022), and EFNB1 (Wieland et al., 2004), and transcription factors SMAD6 (Calpena et al., 2020), MSX2 (Jabs et al., 1993), and TWIST1 (Ghouzzi et al., 1997; Howard et al., 1997). EFNA4 and EFNB1 encode members of the Ephrin pathway, which is known to regulate the decision between maintaining stemness and differentiating into osteogenic cells (Matsuo and Otaki, 2012). Intriguingly, many of the genes mutated in craniosynostosis are downstream of RUNX2, which is itself mutated in some cases of syndromic craniosynostosis (Jaruga et al., 2016).

Notably, many of these genes function downstream of RUNX2, a master regulator of osteogenesis that integrates FGF, Wnt, and Hedgehog signals to drive osteoblast commitment (Komori, 2022). The critical role of these and related pathways in craniosynostosis is supported by syndromic mutations in other genes of these pathways, including the hedgehog pathway gene SMO (Twigg et al., 2016) and RAB23 (Jenkins et al., 2007), and between MAPK pathway genes FGFRs, IL11RA (Nieminen et al., 2011), ERF (Moortgat et al., 2018), and FGFR1, FGFR2, and FGFR3. IL11RA enhances osteoblast differentiation and bone formation and mitigates osteoclast-induced bone resorption (Nieminen et al., 2011). The roles of other genes mutated in syndromic craniosynostosis are not yet clear but are likely to act through other pathways that influence the decision between stemness and differentiation, including genes HUWE1, TCF12 (Di Rocco et al., 2014), and ZIC1 (Stanton et al., 2022; Twigg et al., 2015). Taken together, these mutations implicate a network of genes essential for SSC function and provide a mechanistic framework for studying the stem cell biology of the cranial sutures.

### 4.3 Key unresolved issues

Although human genetic studies of syndromic craniosynostosis have revealed key pathways involved in osteogenesis and suture function, translating these findings into a coherent model of human cranial SSC biology remains challenging. In humans, pathogenic variants in FGFR2, TWIST1, EFNB1, SMAD6, and related genes implicate stem-cell regulatory pathways. But most studies analyze bulk tissue or osteoblast-biased samples, leaving the SSC-specific consequences unresolved. As a result, it is often unclear whether these mutations act primarily on *bona fide* SSCs or their progeny. Additionally, several foundational aspects of human cranial SSC biology remain unsettled due to major conceptual and methodological uncertainties. Box 2 summarizes the central unresolved controversies that currently limit definitive interpretation of human cranial SSC behavior and disease mechanisms.

Box 2 **Key unresolved controversies about cranial SSCs**

**Identity and Definition:** There is debate about the exact markers and criteria that delineate cranial skeletal stem cells from mesenchymal cells. The use of “SSC and MSC” in the literature can lead to confusion and conflicting results in the literature regarding what constitutes a “pure” cranial SSC population.

**Niche Localization:** The proportional contribution of each niche to cranial bone growth and homeostasis is not fully resolved. Recent studies have shown that the cranial suture mesenchyme is a major stem cell niche. However, other potential sources such as the periosteum, dura mater, and diploic bone marrow could still contain progenitor cells and remain under investigation.

**Functional Heterogeneity:** Subpopulations of cranial SSCs exhibit clonal and functional heterogeneity. It is unclear which subpopulations are the most therapeutically relevant due to variable differentiation potential and immunomodulatory properties.

**Translational Potential and Safety:** There is still uncertainty about the most optimal strategies for isolating, expanding, and using cranial SSCs in clinical settings. Further, it is debatable whether cranial SSCs will be able to reliably regenerate complex craniofacial structures in humans.

## 5 Stem cell dysfunction as a driver of suture fusion

As outlined in Box 2, there are multiple unresolved questions about SSCs and their behavior. Despite these uncertainties, converging functional and genetic evidence suggests a model in which disruption of SSC maintenance plays a central role in suture pathology. Single-cell and marker-based analyses of human craniosynostosis specimens demonstrate that disruption of the skeletal stem cell (SSC) niche is associated with pathological suture fusion.

Prospective FACS-based isolation has identified a significant reduction in the representation of skeletal stem and progenitor cells in fusing sutures compared to patent sutures in nonsyndromic craniosynostosis patients, indicating depletion or imbalance of the stem cell pool at sites of premature fusion (Menon et al., 2021). Further, AXIN2 expression, one marker of Wnt-responsive suture stem cells, is diminished in cells isolated from prematurely fused sutures (Di Pietro et al., 2020). At the transcriptional level, single-cell RNA sequencing of human coronal sutures reveals distinct gene expression programs in cells derived from physiologically closing versus persistently patent sutures, highlighting substantial heterogeneity among suture-resident stem and progenitor populations (Menon et al., 2021; Farmer et al., 2021). Functional abnormalities show that cells isolated from fused sutures exhibit pathological overactivation of autophagy, consistent with accelerated or dysregulated ossification (Qiu et al., 2018). While multiple lines of human evidence support an association between SSC dysfunction and suture fusion, it is important to note that these findings remain correlative and do not provide the temporal or causal resolution required to establish SSC dysfunction as the initiating driver of suture fusion.

In contrast, murine genetic models provide strong mechanistic evidence that disruption of SSC maintenance is sufficient to induce suture pathology. Lineage-tracing studies demonstrate that depletion or premature differentiation of Gli1^+^, Axin2^+^, or Prx1^+^ suture stem cell populations precedes fusion and leads to loss of the undifferentiated mesenchyme necessary for controlled intramembranous ossification (Zhao et al., 2015; Maruyama et al., 2016). These findings suggest that SSC maintenance failure is a primary causal event in murine models and a plausible, but not yet proven, driver in human suture fusion.

Definitely determining whether SSC dysfunction is a primary causal event in human suture fusion or instead a secondary consequence remains a critical objective for the field. As outlined in Box 2, resolving this distinction will require approaches that directly test whether restoring or preserving SSC maintenance can prevent or reverse fusion in human-relevant systems, and rigorously exclude alternative mechanisms. Clarifying the causal role of SSC dysfunction is essential for refining mechanistic models of craniosynostosis and for developing therapies that address the underlying biological defect, rather than merely treating the structural consequences of premature suture fusion.

## 6 Therapeutic applications of SSCs

Current treatment of craniosynostosis relies almost exclusively on surgical reconstruction to correct cranial shape and mitigate the risk of neurologic impairment (Bestances et al., 2025). However, despite refinements in surgical technique, postoperative challenges remain, particularly in cases requiring extensive bone remodeling or revision procedures (Magge et al., 2024). A critical limitation of existing reconstructive strategies is the lack of reliable, autologous bone regeneration in areas of craniectomy or suturectomy. Grafts harvested from the patient’s own bone are limited in supply, and synthetic materials such as titanium or polyetheretherketone (PEEK) lack osteointegration, posing long-term risks of exposure, infection, and mechanical failure (Alotaibi et al., 2020; Eppley et al., 2004; Magge et al., 2024; Thien et al., 2015). In this context, SSCs offer a promising avenue for biologically integrated, autologous bone regeneration that could substantially improve reconstructive outcomes in craniosynostosis. As our understanding of SSC identity, localization, and signaling regulation has expanded, so too have the prospects for their clinical deployment (Li et al., 2022).

Although efforts to translate skeletal stem cell biology into craniofacial regeneration have advanced rapidly, most strategies rely on assumptions that do not fully reflect the unique properties of human calvarial SSCs. Many preclinical studies use heterogeneous cells isolated from marrow, adipose tissue, or long-bone periosteum and implicitly treat them as functionally equivalent to neural crest–derived cranial SSCs. It is important to note that this equivalence is largely untested. Moreover, widely used rodent calvarial defect models have thin bones, robust vascularity, and rapid intrinsic healing. These models cannot adequately capture the difficulty of regenerating thick, vascularized human cranial bone. These limitations do not diminish the promise of SSC-based therapeutics but highlight the need for mechanistically tailored approaches that account for the biology, lineage origin, and niche requirements of true cranial SSCs.

### 6.1 Scaffold-based SSC delivery and bone regeneration

Biomaterial scaffolds provide a structural and biochemical microenvironment conducive to SSC survival, proliferation, and differentiation. Osteoconductive scaffolds composed of collagen, hydroxyapatite, or β-tricalcium phosphate (β-TCP) have been shown to support cranial bone regeneration in preclinical models (Geng et al., 2021; Torres-Guzman et al., 2023). When seeded with cells, these constructs can repair critical-sized calvarial defects via intramembranous ossification, especially when augmented with osteoinductive molecules such as BMP-2. Incorporation of angiogenic cues such as vascular endothelial growth factor (VEGF) further enhances integration and long-term viability by promoting neovascularization.

Calvarial bone regeneration requires not only osteogenesis but also adequate vascularization to support cellular viability and metabolic demands (Patel et al., 2008). Delivery of VEGF in combination with SSCs has been shown to significantly enhance bone repair in large-animal models (Dreyer et al., 2021), resulting in improved vessel density and bone volume. Future directions include developing multicellular constructs incorporating both osteogenic and endothelial progenitors, thereby recapitulating the coupled osteoangiogenic niche of native bone (Zheng et al., 2024).

### 6.2 Pharmacological and exosome-based modulation of SSCs

Small molecule modulation of key signaling pathways represents a promising cell-free approach to activate endogenous SSCs. Agonists of Wnt signaling, such as lithium chloride or GSK-3β inhibitors, have been shown to stimulate osteogenic differentiation in calvarial progenitor populations (Clément-Lacroix et al., 2005; Xia et al., 2017). Similarly, controlled delivery of BMPs has demonstrated efficacy in promoting calvarial bone formation, though supraphysiologic BMP levels carry risks of ectopic ossification and inflammation (Surisaeng et al., 2025). Targeting these pathways with temporally regulated or localized delivery systems could enhance safety and efficacy in clinical contexts.

Further, recent work has explored the use of SSC-derived extracellular vesicles (EVs) as a cell-free strategy to promote bone healing. EVs derived from mesenchymal or skeletal progenitor cells carry osteogenic microRNAs, growth factors, and matrix-modulating enzymes that enhance bone repair (Al-Sharabi et al., 2024). These vesicles avoid some of the risks associated with live cell therapies, such as immune rejection or tumorigenicity, and may be particularly suitable for pediatric applications (Liu et al., 2023).

### 6.3 Genetic engineering and stem cell reprogramming

Genome-editing platforms such as CRISPR/Cas9 enable modification of SSC fate at the genetic level. For example, correction of pathogenic mutations in FGFR2 or SMAD6 could restore balanced osteogenesis in patient-derived SSCs. Although still largely preclinical, studies in murine SSCs have demonstrated that modulation of transcriptional regulators, such as Axin2 and Twist1, can alter osteogenic commitment and repair capacity (Holmes et al., 2021; Timberlake et al., 2016; Yue et al., 2024). The application of these technologies to human SSCs isolated from the calvarium may enable personalized, autologous regenerative therapies.

Together, these emerging strategies underscore the translational potential of cranial SSCs in both congenital and acquired craniofacial disorders. While most studies to date remain in the preclinical phase, the development of standardized isolation protocols, functional assays, and delivery systems for human calvarial SSCs will be essential to advance clinical implementation. Harnessing these stem cells not only offers a means of regenerating calvarial bone but also opens new avenues for preventing disease recurrence and minimizing invasive surgical interventions.

The key advantages, limitations, and anticipated translational challenges of each strategy are summarized in Table 1. Addressing these gaps will be essential for advancing SSC-based interventions from concept to clinically actionable therapies.

**TABLE 1:** Therapeutic applications of SSCs.

Strategy		Representative applications	Mechanism	Key advantages	Major limitations
6.1	Scaffold-based cell delivery	1. Collagen, hydroxyapatite, or β-TCP scaffolds with SSCs 2. BMP-2/VEGF-loaded constructs for calvarial defects	Provides osteo-conductive structure and supports stem cell survival and osteogenic differentiation	1. Potential for patient-specific defect filling 2. Pre-existing well characterized scaffold materials	1. Requires reliable SSC sourcing and expansion 2. Risk of ectopic ossification with high BMP doses 3. Integration may differ between children and adults
6.2	Endogenous SSC activation via small molecules or growth factors	1. Wnt agonists such as GSK-3β inhibitors 2. BMP delivery or VEGF co-delivery	Modulates signaling pathways to enhance proliferation and differentiation	1. Avoids *ex vivo* cell manipulation 2. Potentially simpler manufacturing and regulation	1. Potential for off-target effects like ectopic bone or dysregulated remodeling 2. Need for precise spatial and temporal control of dosing
6.2	Extracellular vesicle-based therapies	MSC- or SSC-derived EVs delivering osteogenic miRNAs and growth factors	Paracrine modulation of SSC behavior and niche environment	1. Cell-free therapy with low immunogenicity 2. Favorable safety profile for pediatric patients	1. EV cargo heterogeneity 2. Challenges in large-scale production, characterization, and standardization
6.3	Gene editing and SSC reprogramming	1. CRISPR/Cas9 correction of FGFR2 or SMAD6 mutations in patient-derived SSCs 2. Modulation of Axin2 or Twist1 in preclinical models	Directly corrects or rewires upstream regulatory programs controlling SSC fate	1. Potential for durable, mutation level correction 2. Opportunities for personalized therapy options	1. Currently limited to pre-clinical models with a poor translatability outlook 2. Concerns regarding off-target effects, long-term safety, cost, regulatory issues, and ethics

### 6.4 Translational bottlenecks and prioritization of SSC-based strategies

A wide range of SSC-based and SSC-informed therapeutic strategies has been proposed (Table 1). However, these approaches encounter specific translational barriers, which are further complicated by current methodological limitations (Box 1).

The biggest bottleneck is posed by therapies that require live cells. Challenges include limited access to suitable human cranial tissue, difficulty maintaining cell identity during *ex vivo* expansion, and loss of regulatory cues. Since the defining features of human cranial SSCs cannot yet be functionally resolved with high confidence, all strategies dependent on precise cell identity remain constrained. Further, all gene-editing and potential endogenous reprogramming approaches are currently limited by safety concerns, regulatory complexity, and ethical considerations. Extracellular vesicle-based strategies circumvent some risks associated with live-cell delivery. However, they are hindered by cargo heterogeneity, incomplete mechanistic understanding, and challenges in scalable manufacturing. Lastly, approaches targeting endogenous cell activation require precise spatial and temporal control of signaling pathways to prevent off-target ossification or aberrant remodeling.

Key priorities include (1) developing standardized, relevant metrics for human cranial SSC-enriched populations; (2) establishing culture and biomaterial systems that preserve niche-dependent identity during expansion; and (3) improving our understanding of temporal and spatial cell regulation. Collectively, these efforts will be critical for translating SSC-based concepts into clinically viable therapies.

## 7 Conclusion

Human cranial skeletal stem cells occupy an important role at the intersection of developmental biology and regeneration. Emerging data support the existence of regionally specialized SSC populations within sutures, periosteum, and intracalvarial compartments that sustain ossification and maintain calvarial architecture. These populations differ substantially from human appendicular and murine SSCs, implying that principles derived from long-bone or mouse models cannot be directly applied to the human skull. At the same time, progress in human cranial SSC biology has been constrained by methodological limitations.

Recent work in craniosynostosis has begun to link these stem cell populations to disease. Genetic studies confirm that many syndromic craniosynostosis genes converge on pathways that govern SSC behavior. In addition, murine models demonstrate that depletion or misspecification of suture stem cells is sufficient to precipitate premature fusion. These findings start to reframe craniosynostosis as a disorder of stem cell niche failure rather than simply excessive osteogenesis. Yet, in human tissues, it remains unresolved whether SSC depletion, altered lineage bias, or secondary microenvironmental insults are the dominant drivers of pathology. Clarifying these relationships is essential both for refining disease models and for identifying the most rational points of therapeutic intervention.

Therapeutically, skeletal stem cells offer a compelling platform for craniofacial reconstruction as summarized in Table 1. Scaffold-based approaches have shown promise in supporting SSC-mediated bone regeneration by providing both structural and biochemical cues that promote osteogenic differentiation and vascular integration. Strategies that incorporate growth factors, small molecules, or extracellular vesicles further expand the range of tools available to enhance SSC function and repair capacity. Gene-editing technologies and stem cell reprogramming approaches, although still in preclinical stages, may enable correction of pathogenic mutations and restoration of normal SSC behavior in disease settings.

Moving forward, we propose that the most important priorities are (1) defining robust, human-specific marker sets that distinguish long-lived SSCs from transient progenitors, (2) integrating spatial transcriptomics and multi-omic profiling to study SSC behavior *in situ*, and (3) engineering scaffolds and delivery systems that recreate appropriate mechanical and vascular needs. Ultimately, resolving these outstanding questions will be necessary to move beyond descriptive frameworks and toward precise, mechanism-based therapies that leverage cranial SSCs to restore developmental signaling, regenerate bone, and prevent pathologic suture fusion.
